# Supplementary material for: IS481EU Shows a New Connection between Eukaryotic and Prokaryotic DNA Transposons
Source: Biology (Basel). 2023 Feb 25;12(3):365. doi: 10.3390/biology12030365 (PMC10045372; doi:10.3390/biology12030365)
Supplement: Supplementary file 1 [file biology-12-00365-s001.zip › SupplementaryMaterials/FigureS1.pdf]

Figure S1. Multiple alignment of DDD/E transposase cores. The three catalytic residues are highlighted in red, while other conserved residues are in green or yellow.

```
Polinton/Polintovirus/Maverick (Eukaryotes)
Polinton-1_TV          RPSFAPYPYSYEIDHLEYS------------------KGNVTYLFA--ININTRYLYCIP
Polinton-1_SM          YKTM-GVDDLWQMDLMEMIPFSKI-------------NKGYKYILTC-VDIFSRFARAIP
Polinton-1_PI          IASH-NPNS-WQIDLA---------------------FWKKRPILTA-ININSRLGYAKL
Polinton-1_DR          VVVY-GMDTQFQADLVDMTAYST--E-----------NDGNKFMLTC-IDVFSKYAWSRV
Mavirus_Spezl          NVQQ-------QADVLYLPAD----------------KNGFKYVLVV-AD-YNRKIGAYP
Tlr1                   IPLS-APQGSFQADLTFYEQFKRQ-------------NHGYSILLTI-IEINTRFAYVFP
LTR retrotransposon (Eukaryotes)
COPIA_DM               THIK-RPLFVVHSDVCGPITPVT--------------LDDKNYFVIF-VDQFTHYCVTYL
BEL                    VQAS-RCFQHTGLDYAGPIAIKESKGRTP--------RIGKAWFSIF-VCLTTKALHIEV
GYPSY                  PIPS-YTGEMVHIDIF---------------------STDRKLFLTC-IDKFSKYAIVQP
Troyka-1_NV            DSPD-AIGVSFAADVI---------------------RRSRQFILIV-RECVTSYTTSLL
Retrovirus (Eukaryotes)
ERV1-1_XT              PTTP-YPFHTIALDFVEMTP-----------------SEGKKYILVI-IDTFSGWIEAFP
ERV2-1_CLan            PRGL-CPNARWQMDVTHFPP-----------------FGKLKYIHVT-KDTFSGATMAIA
ERV3-1_AMi             PRGH-GPAEKWQVDYIGPLPA----------------INHYRYALTA-IDTFSGYGIAVP
SFV1-10_AMi            PSQF-QAGKVWQIDLLGPLPG----------------SKPPNKGLVI-VDLGTRQLQVIP
HTLV-1                 RRGL-LPNHIWQGDITHFK------------------YKNTLYRLHVWVDTFSGAISATQ
HIV-1                  HGQVDCSPGIWQLDCT---------------------HLEGKVILVA-VHVASGYIEAEV
CoeEFV                 RGAL---FDKIFLDFVGPLPR----------------SNGYTAILIL-VESLSSFTWLLP
Loki-1_PM              RGTD-YPWSNLQIDWIGPVNRS---------------TKGNKYMLTV-TCAFSKWIECLP
Ginger (Eukaryotes)
Ginger1-1_AP           HVPK-KVFKQIGVDLITLPE-----------------VNNLRYVVVV-VCYFSKWCEAKA
Ginger1-9_HM           KVKD-EVWSTVGIDLIGPLPLT---------------EKGNKYIITA-TCLFSKWPEAAS
Ginger1-2_OT           IYSS-RPLERIQIDTTDIQHL----------------NSKYPYLLCI-VDHFSKFAQAYT
Ginger2-1_LS           IVEK-DFLERVQVDLIDMRHNP---------------DGEFNHICHF-MDHFSKYHVLFP
Ginger2-1_NV           FQAP-TFLSFIELDLMDFRNTPCKC------------SKRHQWVLNI-IDHHSKYVTCWP
Ginger2-1_CGi          IRAS-SIQERHQIDLVDMSRYTEFYD-----------GQEYRYILSV-IDVFSRYIWLRP
IS481 (Prokaryotes)
IS481_6bp              YEHQ-APGDLLHIDIKKLGRIQRPGHRVTGNRRDTVEGAGWDFVFVA-IDDHARVAFTDI
ISAar27_14bp           FARS-AAMALWQLDAFEFRTH----------------SDQVVTVYQL-IDDATRFDVGSS
ISEc18_2bp             YERP-VPGDRVQMDTCK--------------------IGPGLYQYTS-VDDCTRSRVLRL
ISRm20_5bp             FEKE-APNQLWQMDFKGWVQLA---------------DATLCHPLTV-IDDHSRFVPCLM
ISRpe1_3bp             TETQ-HPGYLGCQDTYYVGNFK---------------GIGKVYSQVF-IDSYTRVADAKL
ISSpal1_6bp            FKD--YEIGYFHIDIAELRY-----------------EGGKAFLYVA-VDRTSKLVFARI
ISChy3_6bp             FAKE-QRNRLWQADIKYGPYLPHPKNPK---------RKVRTYLVAF-IDDATRLLCHGE
ISA0963-1_15bp         YERT-HSMSLWQGDWK---------------------KLGEKWIIAF-MDDASRFITCYG
IS481EU/Banshee (Eukaryotes)
IS481EU-1_TV_15bp      YWAL-SPNLIWHADVHYFR------------------GVRGQYIYGI-IDDFSRKVLACI
IS481EU-3_TV_4bp       YEAV-YINLIWHVDIHFFK------------------HRQGEYLYAV-IDDKSRKILAWA
IS481EU-1_HisMel_15bp  FVAK-YANQLWHADIHYVTI-----------------EGEQFYLLGF-IDDRSRFLIYYE
IS3EU/GingerRoot (Eukaryotes)
IS3EU-1_SK             YNVM-GPNHLWHVDTNHKL------------------IRWHMIIFGC-IDGFSRLPITLA
IS3EU-3_DR             YSVP-APLSLVHVDTNHKL------------------ITFNIVIFGG-IDGFSRKILYLD
IS3EU-4_CGi            YAAP-GPNFVWHIDGYDKL------------------KPFGFAIHGA-IDGFSRRIMWLE
SL_GR-020              YNVI-EPMGLWHMDSYDKLA-----------------VYGFRF-HGI-IDGATRFIVACS
SL_GR-005              FSRL-EPMELWAMDSYDKL------------------SMYGIRIHGA-IDASSHCVVYMV
IS3 (Prokaryotes)
IS407_4bp              ALPG-APNEVWSIDFVMDALS----------------NGRRVKCLTV-VDDFTKEAVDIV
IS2_5bp                VAVK-ESNQRWCSDGFEFCC-----------------DNGERLRVTFALDCCDREALHWA
IS1141                 LVAF-EPNQVWSWDITKLRGPA---------------KWSWYYLYVI-LDIFSRYVVGWM
IS3_3bp                FYAS-GPNQKWAGDITYLRT-----------------DEGWLYLAVV-IDLWSRAVIGWS
IS51_3bp               FVAE-CPNALWVSDFTYVST-----------------WQGFVYVAFI-IDVFARFIVGWK
IS120_3bp              LKID-HPNQVWSIDVTYCRM-----------------KRGFMYMVAI-IDWYSRYIVGFE
IS150_3bp              FKAT-RPNEKWVTDVTEFAV-----------------NGRKLYLSPV-IDLFNNEVISYS
IS1138_3bp             YNGK--FNNIVATDVTYIPSPKDA-------------INNHVYLSIA-IHHQSKKIINWN
IS1202 (Prokaryotes)
IS1202_27bp            PRKK-FAGELIQMDASPHAWF----------------GPETTNLHLA-IDDASGNILGAY
ISApi2_5bp             YNRD-CYGELIQIDGSHHDWFEG--------------RAPKCCLLVF-IDDATGKLQHLR
ISTde1_17bp            ERRE-CFGQMLQFDGSHHKWFEQ--------------RGPKCCLMNI-VDDATGMTQSFL
                                    *
```

```
Polinton/Polintovirus/Maverick (Eukaryotes)
Polinton-1_TV          VK-GKT-EQETRRAIQ-------YLLDHERVDN----IRGDG-DKG-F------------
Polinton-1_SM          TK-TKS-ANEMAEAIK-EL----FID--GK-PR--N-LQTDL-GKE-F-------YN---
Polinton-1_PI          LR-KKT-AAIVLEALK-A-----FVRLHKV-----DILTSDN-GSE-F-------MN---
Polinton-1_DR          LK-NKS-GVEVTKAFE-SI----LEE-GRV-PR--K-LQTDQ-GKE-F-------FN---
Mavirus_Spezl          LK-TLN-GEEIVKAFK-EI----YKN-DGLTPP--AIIQFDN-GPE-F-------KN---
Tlr1                   LK-DKS-AQSILECLK-EL----YRT-EKD-NF--KSISSDE-GSE-F-------DN---
LTR retrotransposon (Eukaryotes)
COPIA_DM               IKYKSD-VFSMFQDFV-AK----SEAHFNL-KV--VYLYIDN-GRE-Y-------LS---
BEL                    VS-ELT-TQAFIAAFQ-RF----IAR-RAK-PT--D-LYSDN-GTT-FHGGKKTLDDMRR
GYPSY                  VV-SRT-IVDITAPLL-QII-------NLF-PNIKT-VYCDN-EPA-F-------NS---
Troyka-1_NV            LT-NEQ-HSTLRDALI-RLCVQ-MRPLDG--PV--AIIRTDP-APG-F-------QA---
Retrovirus (Eukaryotes)
ERV1-1_XT              SA-KAD-AIAVAKPLT-RE----IIPRWGI-PE--K-IISDN-GSH-F-------VN---
ERV2-1_CLan            MT-GET-AQHASRALE-KA----ILM-LGV-PW--D-LKTDN-GPC-Y-------TS---
ERV3-1_AMi             VR-KAD-ASSTIRVLE-AH----LVHIHGI-PR--E-ISSDQ-GTH-F-------TA---
SFV1-10_AMi            LR-KSN-ATAVVSALT-AA----FAA-WQP-PH--E-IQSDG-GPP-F-------NS---
HTLV-1                 KR-KET-SSEAISSLL-QA----IAY-LGK-PS--Y-INTDN-GPA-Y-------IS---
HIV-1                  IP-AET-GQETAYFLL-KL--------AGRWPV--KVIHTDN-GSN-F-------TS---
CoeEFV                 CR-DQS-ASTTVTALS-AR----FFP-ASIEPK--C-FHSDQGGGD-F-------TS---
Loki-1_PM              AP-NNT-AETTAILLI-NQI---FSR-WGL-PS--T-IDSDK-GSH-F-------TA---
Ginger (Eukaryotes)
Ginger1-1_AP           LM-DKT-AESVAKFLY-DD----ICR-HGC-PE--I-QISDQ-GRE-F-------VN---
Ginger1-9_HM           LS-DKT-ATSAAEFLY-TC----FTR-HGCCEV----QISDQ-GRE-F-------VN---
Ginger1-2_OT           LK-NKS-AETVLKNIK-K-----YLSQFGI-PY--I-IQSDN-GLE-F-------CN---
Ginger2-1_LS           LK-PKS-AVEVAGLIE-ER----VLAYFGP-PK--L-FHSDN-GRE-F-------VN---
Ginger2-1_NV           LK-GKN-AVEVLQGLK-N-----YCYSFGF-PK--K-IVCDN-GTE-F-------SN---
Ginger2-1_CGi          LR-SKE-STEVADALE-SI----YQS-EGP-PK--I-IQCDN-GRE-F-------HG---
IS481 (Prokaryotes)
IS481_6bp              HP-DER-FPSAVQFLK-DAV-AYYQR-LGV-TI--QRLLTDN-GSA-F-------RS---
ISAar27_14bp           AYARHENSGDAQQVLA-RA----IND-YGP-PK--E-VLSDN-SKA-F-------NQLRG
ISEc18_2bp             YS-RRT-AANTLDFI--DC----VIEEMPF-PI--QRLQTDR-GRE-F-------FA---
ISRm20_5bp             ACADQR-GATVRGHLE-RT----FRR-YGL-PD--A-MFVDN-GAP-W-------GDPSG
ISRpe1_3bp             YT-DKT-ALTAADMLN-DRVLPWYET-QGI-PI--LRILTDR-GSE-Y-------KG---
ISSpal1_6bp            YR-KAT-KLAAAAFLK-------VLV-KTV-PYRIHTVLTDN-GVQ-FVQPQRGQSR---
ISChy3_6bp             FYLDQK-RPVLEDCFR-KA----ILK-RGI-PD--A-VYVDN-GKI-F-------VS---
ISA0963-1_15bp         VFDSAT-TENTIKVFK-KG----FAE-YGI-PD--E-ILTDH-GTQ-FVAAKSREKAR--
IS481EU/Banshee (Eukaryotes)
IS481EU-1_TV_15bp      QIPDML-AATTANVAA-SA----FIE-FGA-PY--C-FWTDN-GSE---------NR---
IS481EU-3_TV_4bp       HLETKE-AAQTSRVID-QC----FQK-YGK-PF--A-IWSDN-GTENF------------
IS481EU-1_HisMel_15bp  VLSSKT-SEACATALI-KA----LGSVVHR-PK--M-LTIDN-GGE-F-------TG---
IS3EU/GingerRoot (Eukaryotes)
IS3EU-1_SK             CTDNNQ-ASTLFKCFM-SG----VEN-YGL-PS--R-VRSDM-GLE---------NV---
IS3EU-3_DR             AAENNK-ASTAFLFFL-EG----VHK-HGW-PS--R-VRGDQ-GVE---------NV---
IS3EU-4_CGi            VGPSNNNPKIVSRYFL-ET----IQQLGGC-PN--I-CRCDL-GTE-N------------
SL_GR-020              VALNKR-PVTIFNNYA-RA----VKK-YGH-PY--R-LRADA-ARE--------------
SL_GR-005              LAMDKR-ATTIYRAFS-AA----TAL-FGR-PR--R-VRSDC-AVE---------HE---
IS3 (Prokaryotes)
IS407_4bp              VDHGIS-GLYVARALD-R-----AARFRGY-PK--A-VRTDQ-GPE-F-------TS---
IS2_5bp                VTTGGFNSETVQDVML-GAVERRFGNDLPSSPV--E-WLTDN-GSC-Y-------RA---
IS1141                 VA-SRE-SAALAEVLIRQTC---AKQDIGR-DR--LTIHADR-GSS-M-------TS---
IS3_3bp                MS-PRM-TAQLACDAL-QMA---LWR-RKR-PRNVI-VHTDR-GGQ-Y-------CS---
IS51_3bp               VSSSAR-TDFVLDALEQAL----YAR-RPV-KQGGLIHHSDR-GVQ-Y-------VS---
IS120_3bp              LSNTLD-KTFVIEAIQ-KA----IKR-YGK-PE--I-MNSDQ-GSQ-F-------TS---
IS150_3bp              LS-ERP-VMNMVENML-DQAFKKLNP-HEH-PV----LHSDQ-GWQ-Y-------RM---
IS1138_3bp             LSKRND-VKLVLDHIS-------KIK-FDK-EW--I-IHSDH-GSQ-Y-------SS---
IS1202 (Prokaryotes)
IS1202_27bp            FDKQET-LNAYYHVLE-QI----LAN-HGI-PL--Q-MKTDK-RTV-FTYQASNSKKMED
ISApi2_5bp             FCASESAFDYMISTRL-------YIEQHGK-PL--A-FYSDK-HSV-F-------RVNQS
ISTde1_17bp            TE-QET-TEAAMRLLW-G-----WIDCHGI-PQ--A-VCCDK-KNA-Y-------VITRE
```

Polinton/Polintovirus/Maverick (Eukaryotes)

```
Polinton-1_TV          -------------QAAMTH---YFPQINF---YFSSSPYTFHNKIVDAVMRTLRDAL-GV
Polinton-1_SM          -------------SKVRE----IIKGI-----NHYSVFSQFKAAHVERFNRTLRDRL---
Polinton-1_PI          -------------FQAQE----LFKSKTIEH-YNNEPGDHGTMGKIERFNRTLKQRL---
Polinton-1_DR          -------------KHFQD----LMKKYDI---NHFATATDLKASVVERFNRTLKSRM---
Mavirus_Spezl          -------------KNVVD----YFKSKNSSI-KYTKPYRSRQNGLVENINFIIGKLVGVY
Tlr1                   -------------NLVKN----FLDQNNIKYIFFNKQTNPNVTSLIERFNRTIRDKI---
```

LTR retrotransposon (Eukaryotes)

```
COPIA_DM               -------------NEMRQ----FCVKKGISY-HLTVPHTPQLNGVSERMIRTITEKA---
BEL                    LAIQQAKD-----EELAG----FFANEGISW-HFIPPSAPHFGGMWEAGVRSIKLHM---
GYPSY                  -------------ETVTSM---LKNSFGIDI-VNAPPLHSSSNGQVERFHSTLAEIA---
Troyka-1_NV            -------------LKEDK----LLQHHRLVL-EIGRAKNVNKNPVAEKAVQELEREI---
```

Retrovirus (Eukaryotes)

```
ERV1-1_XT              -------------EVIKQ----LTTSLGIQV-RHHCSYHPQSAGKVERANGVLKNRL---
ERV2-1_CLan            -------------QTFKN----TCAKYNITL-HHGIPYNPQGQAIIERTHQTLKTLL---
ERV3-1_AMi             -------------LLTQQ----WAAGLGIAW-TFHVPYHPQAAGLIERWNGQLKQLL---
SFV1-10_AMi            -------------NALDK----FARLHNVAW-HLHLPYHPQSNGVVERHIGLYKEQL---
HTLV-1                 -------------QDFLN----MCTSLAIRH-TTHVPYNPTSSGLVERSNGILKTLL---
HIV-1                  -------------AAVKA----ACWWANVQQ-EFGIPYNPQSQGVVESMNKELKKII---
CoeEFV                 -------------QLFKK----MCSERNIRV-EYSTPHHPQSAGVVERKNRGLKAAL---
Loki-1_PM              -------------EVITE----VWKMLGVRR-QLHVAYHPQSSGQVERANRTIVSLL---
```

Ginger (Eukaryotes)

```
Ginger1-1_AP           -------------KLSDE----LFRLTGTQQ-RVTSPYHPQANGLVERLNRTLKTSL---
Ginger1-9_HM           -------------EVNHE----LNKMMGTKC-NVTSAYHPQSNGEDERFNQTLQRQL---
Ginger1-2_OT           -------------KLMEEL---IQNHQNIQH-ITSRPYNPRAQGCVEAFNKTIKKEL---
Ginger2-1_LS           -------------QIIRAL---FSSWGGVTF-VNGRPRHSQSQGLVERGNRTIEQKL---
Ginger2-1_NV           -------------AMLNS----FCEENNIKI-CHGSPRTPTTQGLVERSNRTWKEDM---
Ginger2-1_CGi          -------------TVTQ-----LAEVLGCQI-INSRPYYPQSQGKIESSHKSWKSKI---
```

IS481 (Prokaryotes)

```
IS481_6bp              -------------RAFAA----LCHELGIKH-RFTRPYRPQTNGKAERFIQSALREW---
ISAar27_14bp           GTI----------GIVEA----YLASQGTMP-ITGLPGRPTTQGKNERSHQTLQQFL---
ISEc18_2bp             -------------VKVQE----KLKEYSIKF-RPNKPASPHLNGKVERSQKTDKAEF---
ISRm20_5bp             EGW----------TGLGV----WLLKLGVAL-LHSRPYHPQSRGKNERFHRTLKAEV---
ISRpe1_3bp             -------------NIEHHAFELFLSIEGIEH-TTTKAYSPQTNGMCERFNKTMKQEFFDT
ISSpal1_6bp            -------------QWLIHIFERVCLENGIEH-RLTKPYHPWTNGQAERMVRTIKEAT---
ISChy3_6bp             -------------RWFRL----GCARLGIRP-INTKPYSPESKGKIERFNRTVESFI--A
ISA0963-1_15bp         -------------HRFRE----FLAENGVRH-ILARVNHPQTNGKIERFFGLMEQKL---
```

IS481EU/Banshee (Eukaryotes)

```
IS481EU-1_TV_15bp      -------------GAFQD----LLDLWGVQW-RHTDSHSPYQNGKIERFWPTLERCQ---
IS481EU-3_TV_4bp       -------------GVFRN----LLQNANVQS-ITILPHCPYMNGKIERFWQNVE------
IS481EU-1_HisMel_15bp  -------------NPFQF----ILQLYGIED-YRTHPYTPEENGKIERFWLTIERAK---
```

IS3EU/GingerRoot (Eukaryotes)

```
IS3EU-1_SK             -------------HIADY----MISKRGENR-GSMITGRSVHNQRIERLWRDVYEGVLSL
IS3EU-3_DR             -------------DIARC----MFSVRGTGR-GSFIAGKSVHNQRIERLWCDVWSAVTSK
IS3EU-4_CGi            -------------KELEEIQVLLHTLNDQEYENCFIYGRSTSNQRIEAWWSILRRQAADW
SL_GR-020              -------------HNLIE----LDMEDAWPGRAAFLKGPSTHNQRIEHQWRDFFEKF---
SL_GR-005              -------------LVAQD----MERHWPNAPKPPFITGSSTHNVKIEAFWRHLYEKV---
```

IS3 (Prokaryotes)

```
IS407_4bp              -------------RALDQ----WAYANGVTL-KLIQAGKPTQNAYIESFNGKFRDEC---
IS2_5bp                -------------NETRQ----FARMLGLEP-KNTAVRSPESNGIAESFVKTIKRDY---
IS1141                 -------------KPVAF----LLADLGVTQ-SHSRPHVSDDNPFSEAQFKTLKY-----
IS3_3bp                -------------ADYQA----QLKRHNLRG-SMSAKGCCYDNACVESFFHSLKVEC---
IS51_3bp               -------------IRYTE----RLVEAGIEP-SVGSVGDSYDNALAETINGLYKAEV---
IS120_3bp              -------------DDYIN----LLKNNGIKI-SMDGKGRALDNQRIERFFRSYKWEK---
IS150_3bp              -------------RRYQN----ILKEHGIKQ-SMSAKGNCLDNAVVECFFGTLKSEC---
IS1138_3bp             -------------NQYSE----IIKENNGII-SMSRIANSLNNREAEYFFSNIKS-----
```

IS1202 (Prokaryotes)

```
IS1202_27bp            DSY----------TQFGY----ACHQLGI---LLETTSIPQAKGRVERLNQTLQSRL---
ISApi2_5bp             SKKDTKI------TQFGR----VLSTLNIDI-IFA--NSPQAKGRVERANRTLQDRL---
ISTde1_17bp            PTMSEIIKNVRPKTPFQK----ACEKLGIQI-IVA--HSAQSKGRVERNHSVYQDRF---
```

Polinton/Polintovirus/Maverick (Eukaryotes)

```
Polinton-1_TV          NGQIYWDGNHDS--------------IIQQLVYY------YNNT----WHRT----IN--
Polinton-1_SM          -KKYFVYKGNKT---------WIN--VLQKAIYS------YNYS----PHRG----LN--
Polinton-1_PI          ---TKMSPKRIT----------QK--LITDVIEN------YNTT----FHRS----IG--
Polinton-1_DR          -WRFLTATNSRR---------YID--VLQDIMTG------YNNS----YHKT----IK--
Mavirus_Spezl          QNKGMLKNKEFK---------WVD--LLPDIVRV------YNKS----QENI--------
Tlr1                   -SKYQSFHKQKN---------FID--ELQKLVKT------YNNT----IHSQ----IN--
```

LTR retrotransposon (Eukaryotes)

```
COPIA_DM               --RTMVSGAKLDKSF------WGE--AVLTATYL------INRI----PSRA----LV--
BEL                    ---KRILGSKAL---------TFE--ELSTVLTQ------IEAILNSRPLCP----TGDN
GYPSY                  ---RCLKLDKKTND-------TVE--LILRATIE------YNKT----VHSV----TR--
Troyka-1_NV            -----LQLDPLGG--------PVSEVALAVATAN------LNAR---IRLRG----LS--
```

Retrovirus (Eukaryotes)

```
ERV1-1_XT              --SKTMNQTGKS---------WMW--CLPIVLLN------MRIT---PKPKG----LS--
ERV2-1_CLan            --KKEGELHPRS---------TPD-ELLTKALIT------INLL----TFDE----KG--
ERV3-1_AMi             --TKTLGPTLQG---------WPS--QLPKTVAT------LNNR---ELVRG--------
SFV1-10_AMi            --RLRGGGTYKN---------WTK--HNHDVLIS------LNTA----KPLW----DE--
HTLV-1                 --YKYFTDKPDLP--------MDN--ALSIALWT------INHL----NVLT----NCHK
HIV-1                  ---GQVREQAEH---------LKT--AVQMAVFI------HNFK----RKGG----IG--
CoeEFV                 --TKLVRNRPRK---------WFQ--VLDIVQTG------LNNT----PIAR----NEHG
Loki-1_PM              --KKYVSTTGRD---------WDT--KLPLVLMA------MRAT----PNRA----TG--
```

Ginger (Eukaryotes)

```
Ginger1-1_AP           -------LKVFK---------WPD--ILQGILFA------YRTT----VHCS----TK--
Ginger1-9_HM           --LKYVDEKQNT---------WDL--YIESILFS------YRVS----VQDS----TK--
Ginger1-2_OT           --QNIQSKLDKKFE-------IKN--AIQDFLIH------YNNK----EHST----TK--
Ginger2-1_LS           --AA--MKNDNG-CGADQTYPWSS--WLPRVMVS------LNSE----VQAT----TN--
Ginger2-1_NV           --RAILISKQKSVGE------WCK--ATMEASYT------MNIT----YHRA----IK--
Ginger2-1_CGi          --KYDLLKTIDS-N-------WVR--DLPKYAML------RNEE----YHSS----LK--
```

IS481 (Prokaryotes)

```
IS481_6bp              -AYAHTYQNSQH---------RAD--AMKSWLHH------YNWH---RPHQG----IG--
ISAar27_14bp           --KANRPQNLAD---------VQK--LLRRYREH------YNQR---RPHQS----LN--
ISEc18_2bp             --YATVDLSTDD---------LKE--LLAEWQHY------YNWE---RPHSA----HN--
ISRm20_5bp             -FAFDRFRDLAA---------VQR--AFDAWREL------YNFE---RPHGA----LD--
ISRpe1_3bp             AMRKKIYTDLDD---------LQL--DLDIWLEH------FNNE---RPHSG--------
ISSpal1_6bp            -VKSFHYASIQE---------LRR--HVSDWLIA------YNFA---KQLKA----LK--
ISChy3_6bp             EIELQQPETLAE---------LNQ--AFAVWVEE-----GYNHH----PHSS----LE--
ISA0963-1_15bp         -----------H---------LFD--SLDEFIYW------YNYV---KPHMS----LN--
```

IS481EU/Banshee (Eukaryotes)

```
IS481EU-1_TV_15bp      ---------------------SIQ--AIPAFIWE------YNNN---TPHED----LPIN
IS481EU-3_TV_4bp       -------IEDAT---------WND---IQSLIEN------YNSN----PHTS----LPKN
IS481EU-1_HisMel_15bp  ------GPNVAW---------SIP--KINSIIHE------YNYFLDASKFEK----NS--
```

IS3EU/GingerRoot (Eukaryotes)

```
IS3EU-1_SK             YSQIFYFLEENN---------LLD--PLDEHHIA-ALHYVYMRKINQKLESWRMAWSR--
IS3EU-3_DR             YYEILHAMVDGV---------LDLSNELHLFCVHYTILPRLKSDLKCFIGSW----NNHP
IS3EU-4_CGi            WITFFKDLRDANL--------FNDGDLLQIDCLRYCFMDLLQEELHRIVIQWNQHRIQVK
SL_GR-020              ----------IW---------WYK--YMLDSMVETRMLNVYNPWHLQSLHDV--------
SL_GR-005              ----VWYYKETL---------WRM--CDSGQISL------EDPW-----HRA--------
```

IS3 (Prokaryotes)

```
IS407_4bp              -LNEHWFTTLAH---------ARA--VIAAWRQD------YNEQ---RPHSA----LN--
IS2_5bp                ---ISIMPKPDG---------LTAAKNLAEAFEH------YNEW---HPHSA----LG--
IS1141                 ---RPDFPDRFD---------SIE--AARRHCQI-FFGW-YNDE---HRHTG----LG--
IS3_3bp                -----IHGEHFI---------SRE--IMRATVFN-YIECDYNRW---RRHSW----CG--
IS51_3bp               -------IHRRS---------WPTRGAVELETLK------WVDW---FNHRRLLEPIG--
IS120_3bp              -LYLEECETVQQ---------LRQ--ITKEYVEH------YNHR---RPHQS----LD--
IS150_3bp              -FYLDEFSNISE---------LKD--AVTEYIEY------YNSRRISLKLKG----LT--
IS1138_3bp             ---ECLNDLKIS---------KLSFKELQEIIQN-YIDW-YNNE---RLQSI----LE--
```

IS1202 (Prokaryotes)

```
IS1202_27bp            --PIELERNKIH---------TLE--EANTFLLS------YIQT----FNEQ----FGNK
ISApi2_5bp             --IKEMRLEGIS---------SIA--DANKWLPC-FIEQ-FNRKFAKMAFNS----KD--
ISTde1_17bp            --VKELRLAKIN---------TIE--KANAFLQKEYLPK-INKKFAIAPLDS----QD--
```

**Polinton/Polintovirus/Maverick (Eukaryotes)**	
Polinton-1_TV	------MK-PVE
Polinton-1_SM	-----GMR-PID
Polinton-1_PI	------MT-PNE
Polinton-1_DR	------MR-PID
Mavirus_Spezl	------KP-PDK
Tlr1	------MT-PQK
**LTR retrotransposon (Eukaryotes)**	
COPIA_DM	---DSSKT-PYE
BEL	SLDP--LT-PAH
GYPSY	------ER-PIE
Troyka-1_NV	---------ARE
**Retrovirus (Eukaryotes)**	
ERV1-1_XT	---------PYE
ERV2-1_CLan	------LS-PAH
ERV3-1_AMi	------QS-PMD
SFV1-10_AMi	------AN-PKK
HTLV-1	TRWQLHHS-PRL
HIV-1	-----GYS-AGE
CoeEFV	------AT-PFF
Loki-1_PM	------RT-PFE
**Ginger (Eukaryotes)**	
Ginger1-1_AP	------YS-PFF
Ginger1-9_HM	------QT-PFY
Ginger1-2_OT	------YQ-PRI
Ginger2-1_LS	------ES-PYK
Ginger2-1_NV	------CS-PYE
Ginger2-1_CGi	------AS-PFY
**IS481 (Prokaryotes)**	
IS481_6bp	------RAVPIS
ISAar27_14bp	------QATPQK
ISEc18_2bp	-----GKT-PME
ISRm20_5bp	------HDVPAS
ISRpe1_3bp	-KYCYGKT-PMQ
ISSpa11_6bp	------FRTPYE
ISChy3_6bp	------NETPAN
ISA0963-1_15bp	---FDELETPYQ
**IS481EU/Banshee (Eukaryotes)**	
IS481EU-1_TV_15bp	PLTGTNYT-PNQ
IS481EU-3_TV_4bp	PTLHTWYT-PNK
IS481EU-1_HisMel_15bp	------LTNP--
**IS3EU/GingerRoot (Eukaryotes)**	
IS3EU-1_SK	HRMRTTHSSPFR
IS3EU-3_DR	IRTERNLS-PNQ
IS3EU-4_CGi	KQCCSPKGKPDV
SL_GR-020	------FLPELK
SL_GR-005	-SIVRLLAGELE
**IS3 (Prokaryotes)**	
IS407_4bp	------YLAPSE
IS2_5bp	------YRSPRE
IS1141	-----LHV-PAD
IS3_3bp	-----GLS-PEQ
IS51_3bp	------HIPPVE
IS120_3bp	------YKTPAE
IS150_3bp	---------PIE
IS1138_3bp	-----WKT-PQQ
**IS1202 (Prokaryotes)**	
IS1202_27bp	TKLSVFEEAP--
ISApi2_5bp	-----LHR-PIA
ISTde1_17bp	-----GHA-PCP
